# Car seat tolerance screen failure in the neonatal intensive care unit: clinical, oxygen saturation and heart rate analysis

**DOI:** 10.1038/s41390-025-04524-w

**Published:** 2025-10-27

**Authors:** Britney He, Paige Howard, Sherry Kausch, Karen D. Fairchild

**Affiliations:** 1 School of Medicine, University of Virginia, Charlottesville, VA USA; 2 Department of Pediatrics, University of Virginia, Charlottesville, VA USA

## Abstract

**Background:**

The AAP recommends that infants born <37 weeks gestation or with risk factors for positional cardiorespiratory instability undergo a car seat tolerance screen (CSTS) before discharge. Understanding characteristics of CSTS failure may inform screening practices.

**Objective:**

Compare clinical, oxygen saturation, and heart rate features in infants who failed vs. passed CSTS.

**Methods:**

We retrospectively reviewed CSTS data from a single level IV NICU (2018–2024). Clinical characteristics of infants who failed vs. passed their first CSTS were compared. For a subset with SpO₂ and HR data, we analyzed hypoxemia and bradycardia events the day before and during CSTS.

**Results:**

We analyzed 2861 CSTSs from 2523 infants. First CSTS failure occurred in 111 infants (4.4%), most commonly due to oxygen desaturation in 85%. Of 111 infants who failed, 77% passed a second screen a mean of 1.9 days later, and overall length of stay was not significantly different based on CSTS result. Among 1136 infants with physiologic data, those who failed had more desaturation events the day before CSTS, and deeper and longer desaturations during the CSTS.

**Conclusions:**

CSTS failure was associated with more oxygen desaturations the day prior. Most infants passed a subsequent screen within a short time.

**Impact:**

- This is the largest analysis of NICU car seat tolerance screens (CSTS) using continuous physiologic data.
- Hypoxemia and bradycardia events occurred in some CSTS designated as “passed” but events were deeper and longer in screens designated as “failed”.
- Infants that failed their first CSTS had lower mean SpO_2_ and more desaturation events in the 24 hours before the screen.

## Introduction

A car seat tolerance screen (CSTS) is recommended by the American Academy of Pediatrics prior to hospital discharge of infants born preterm ( < 37 weeks gestation) and those with hypotonia or airway compromise. The rationale is that these infants may have cardiorespiratory instability when seated upright or semi-reclined.^1–3^ Some have challenged the utility of the CSTS, and there is variability in performance and interpretation of the screen.^4,5^ A deeper understanding of the characteristics of car seat-related cardiorespiratory instability, particularly for infants in the Neonatal Intensive Care Unit (NICU), might inform clinical care.

NICU patients commonly experience episodes of oxygen desaturation and bradycardia. For those born very preterm, these events are related to immature control of breathing (central, obstructive, and mixed apnea). As infants approach term corrected age these spells of desaturation and heart rate (HR) deceleration decrease in frequency and severity, but mild self-resolved events may still occur when infants are deemed stable for discharge. Hypotonia in both preterm and term infants may also lead to airway compromise and cardiorespiratory instability when positioned semi-reclined in the car seat. While past reports have described CSTS failure rates, details about oxygenation and HR patterns in failed CSTS are lacking.

We sought to identify clinical characteristics of patients in our NICU who fail the CSTS. Additionally, since our research group routinely archives oxygen saturation data from the pulse oximeter (SpO_2_) and heart rate data from the electrocardiogram (HR) and has developed tools for vital sign analysis,^6–10^ we sought to analyze cardiorespiratory patterns during and prior to the CSTS to better understand how our center identifies CSTS failure.

## Methods

We analyzed CSTS results and outcomes from all infants discharged from 2018 to 2024 from the University of Virginia NICU, a level IV unit that serves as a regional referral center for central and southwest Virginia. All CSTS dates and times were identified through electronic medical record review. The study was approved by the University of Virginia School of Medicine Institutional Review Board with waiver of consent due to the non-interventional and deidentified nature of the work.

### CSTS unit policy and procedures

Infants underwent CSTS in the car seat in which they would be transported home after confirmation of compliance with manufacturer's weight and length guidelines. Per institutional policy, the CSTS was conducted within 48 h of anticipated hospital discharge. Typically, the CSTS was performed at night and began shortly after an infant was fed. The infant was harnessed in their safety seat, and the seat was positioned at the angle at which it would be placed in the car. HR was monitored via electrocardiogram, and SpO_2_ via pulse oximeter. A nurse observed the infant for the duration of the screen and entered HR, SpO_2_ and respiratory rate into the medical record vital sign flow sheet every 15 min. CSTS duration was 120 min or the duration of travel to the patient’s home, whichever was longer. Typically, nurses use bedside monitor alarm parameters to identify events of concern. The alarm threshold for bradycardia in our unit is 90 beats per minute, and the low SpO_2_ alarm is set at 88%. Our unit nursing guidelines for stopping the CSTS include the following: apnea >20 seconds, heart rate <90 beats/minute for > 20 seconds, SpO_2_ < 88% for > 20 seconds with a reliable waveform, and respiratory distress that does not improve with proper positioning. If the CSTS was terminated, the time and reason(s) for screen failure were documented by the nurse entering a brief description in the flowsheet. There was no requirement for the nurse to notify a physician or licensed independent practitioner in real time about a CSTS that was prematurely terminated due to concerning vital signs.

For those infants that failed the first CSTS, timing of a repeat screen was at the discretion of the health care team. For those that failed multiple CSTSs, a car bed was utilized at discharge.

### Data collection

The outcome of the CSTS—pass or fail—and reasons for fail were obtained from nursing documentation. Other variables were extracted from the medical record, including sex, race, ethnicity, gestational age (GA), presence of severe IVH (grade III-IV), postmenstrual age (PMA) at time of first car seat test, and whether the infant required supplemental oxygen at discharge. Requirement for home oxygen was not determined based solely on intermittent desaturation events while in the car seat.

### HR and SpO_2_ metrics

For a subset of patients with available archived data, we analyzed electrocardiogram-derived HR and pulse oximeter-derived SpO_2_. Continuous HR and SpO_2_ data were collected from standard NICU bedside monitors (GE Healthcare, Chicago, IL) using the BedMaster system (Hillrom’s Medical Device Integration Solution, Chicago, IL; formerly Excel Medical, Jupiter, FL). HR and SpO_2_ were collected every 2 s. SpO_2_ was measured with Masimo sensors using the default averaging time of 8 s. HR and SpO_2_ data underwent single-step preprocessing where values representing incontrovertible artifacts (zeros) were removed.

We calculated, in 10-minute non-overlapping windows, the mean and minimum HR and SpO_2_. The 10-minute values were then summarized for each infant over two different time windows - the 24-hour day prior to the CSTS and the duration of the CSTS - using the mean for mean HR and mean SpO_2_ and using the minimum value for the minimum HR and minimum SpO_2_. We also quantified episodes of hypoxemia (SpO_2_ < 90% for at least 10 seconds) and bradycardia (HR < 100 beats/minute for at least 5 seconds) the day before and during the CSTS. This threshold for bradycardia was used because it was consistent with our prior published research on apnea of prematurity. For infants with at least one hypoxemia or bradycardia event during the CSTS, we additionally summarized the number of events, mean duration per event, and total duration of events.

### Statistical analyses

We used Wilcoxon rank sum tests and Fisher's exact or chi-squared tests to compare continuous and categorical variables among infants who passed versus failed their initial CSTS and to compare variables among infants who experienced an episode of hypoxemia or bradycardia. All vital sign analyses were performed with R Statistical Software (v4.4.1).

## Results

During the period of study, 2524 infants underwent CSTS; 2413 (95.6%) passed and 111 (4.4%) failed their first screen. Demographic and clinical characteristics by pass/fail outcome are summarized in Table 1. GA was the same (median 34.4 weeks) and length of stay similar (median 24 versus 22 days, *p* = 0.50) in infants that passed and failed their first CSTS.

**Table 1:** Patient characteristics for total population based on first CSTS pass/fail.

Characteristic	CST Fail N = 111^a^	CST Pass N = 2,413^a^	p-value^b^
Female	46 (41%)	1,060 (44%)	0.6
Race			0.001
Other/Unknown	3 (2.7%)	261 (11%)	
Black	12 (11%)	433 (18%)	
White	96 (86%)	1719 (71%)	
Ethnicity			0.7
Non-Hispanic	102 (92%)	2196 (91%)	
Hispanic	9 (8.1%)	217 (9.0%)	
Gestational Age (weeks)	34.4 (32.0, 37.3)	34.4 (32.0, 36.7)	0.8
Birthweight (kg)	2.37 (1.68, 2.98)	2.19 (1.62, 2.77)	0.3
Oxygen at Discharge	8 (7.3%)	240 (10%)	0.3
PMA at CSTS	38.0 (36.2, 39.4)	37.2 (35.9, 40.2)	0.5
Length of Stay (days)	24 (13, 44)	22 (12, 45)	0.5
Severe IVH (Grade III-IV)	1 (0.9%)	44 (1.8%)	0.7

^a^*n* (%); Median (Q1, Q3).

^b^Pearson’s Chi-squared test; Wilcoxon rank sum test; Fisher’s exact test

*CSTS* car seat tolerance screen, *IVH* intraventricular hemorrhage, *PMA* post-menstrual age.

Continuous SpO_2_ and HR data prior to and during the CSTS were available for 1085 who passed and 51 infants who failed the first screen (45% and 46% of the total cohort, respectively). Demographic and clinical features of these infants are shown in Supplemental Table 1. In this sub-cohort, infants that failed the first CSTS trended toward lower gestational age (33 versus 34 weeks, *p* = 0.056) and had significantly longer length of stay (median 30 versus 19 days, *p* = 0.034).

**Fig. 1:**
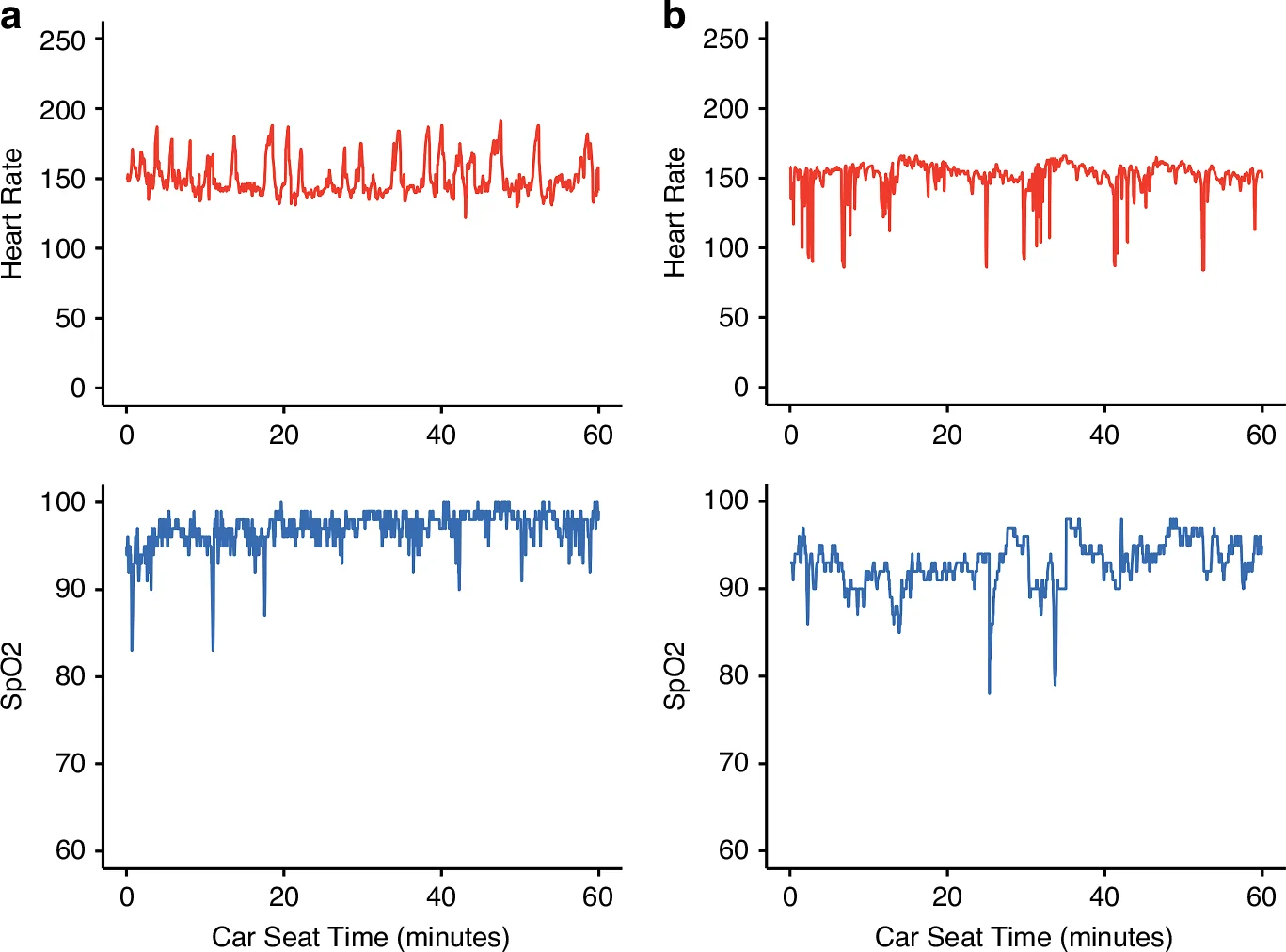
Example of passed and failed CSTS. Time series display of every 2-second heart rate and SpO_2_ for a one-hour span during a CSTS for an infant who passed (**a**) and an infant who failed (**b**). The infant who failed had multiple HR decelerations whereas the infant who passed had accelerations and no decelerations. The infant who failed also had more and longer oxygen desaturation events.

Examples of SpO_2_ and HR in a pass and fail CSTS are shown in Fig. 1. The most common reason for CSTS failure was oxygen desaturation in 85% of cases (Table 2). Ten patients failed due to some combination of tachypnea, tachycardia, increased work of breathing, and/or agitation, which we classified as cardiorespiratory distress. For one infant the reason for failure was documented as positioning issues, and for one, there was no indication in the medical record of the reason for failure.

**Table 2:** Characteristics of CSTS failure and delayed discharge requiring repeat CSTS^a^.

Failed first CSTS	n = 111
Reason for failure	
Desaturation	83 (75)
Bradycardia	5 (4.5)
Desaturation and Bradycardia	11 (9.9)
Cardiorespiratory distress	10 (9.0)
Other	2 (1.8)
Passed second CSTS	86 (77)
Failed second, passed third	18 (16)
Failed more than two, eventually passed	1 (0.9)
Days from first fail to first pass	1.9 (3.0)
Never passed, discharged with car bed	6 (5.4)
**Passed first CSTS, reasons for delayed discharge**	**n** = **130**
Feeding, weight gain	53 (41)
Apnea, brady, desaturation events	38 (29)
Social issues or home equipment	9 (6.9)
Hyperbilirubinemia requiring phototherapy	5 (3.8)
Concern for Infection	13 (8.4)
Other	12 (9.9)

*n* (%) or mean (SD).

^a^unit protocol in the years of the study was to perform the CSTS within 2 days of discharge.

The majority of infants who failed the first CSTS passed a subsequent screen, with a mean interval from fail to pass of 1.9 days. Six infants were discharged in a car bed, representing 0.2% of the total cohort and 5.4% of those that failed the first CSTS. Because of the unit policy of performing the CSTS within 2 days of NICU discharge, 130 infants underwent repeat testing due to delayed discharge, the reasons for which are shown in Table 2. Of these, 4 (3%) failed the repeat CSTS, but all passed a subsequent screen prior to discharge.

Every-2-second SpO_2_ and HR data were available the day before and during the first CSTS for 1136 infants (Table 3). In the day before the CSTS, infants that failed the screen had significantly lower mean SpO_2_ (97.5% versus 98.2%, *p* = 0.006). All infants that failed, and 99% of those that passed, had at least one episode of SpO_2_ < 90% for at least 10 seconds in the day prior to the CSTS. During the CSTS, 49% of infants that passed had at least one event of SpO_2_ < 90%, compared to 88% of those that failed. Mean SpO_2_ during the CSTS was significantly lower for those who failed (93.6% vs 97.5%), as was the minimum SpO_2_ (76% vs. 84%). Bradycardia (HR < 100 beats per minute for at least 5 seconds) occurred during the first CSTS in 29% of those that failed and 8% of those that passed.

**Table 3:** SpO_2_ and HR characteristics in the 24 hours before and during the first CSTS.

	HR/SpO_2_ Characteristic	CSTS Fail N = 51	CSTS Pass N = 1,085	p-value
**Day before first CSTS**	Mean SpO_2_	97.5 (96.3, 98.5)	98.2 (97.1, 98.9)	0.006
	Minimum SpO_2_	65.0 (56.0, 73.0)	67.0 (56.0, 73.0)	0.9
	SpO_2_ < 90%^a^	51 (100%)	1,069 (99%)	>0.9
	Mean HR	154 bpm (147, 160)	153 bpm (145, 159)	0.2
	Minimum HR	81 bpm (67, 96)	87 bpm (70, 102)	0.2
	Bradycardia^b^	32 (63%)	591 (54%)	0.2
**During first CSTS**	Mean SpO_2_	93.6 (91.6, 95.9)	97.5 (95.8, 98.7)	<0.001
	Minimum SpO_2_	76.0 (68.0, 81.0)	84.0 (78.0, 89.0)	<0.001
	SpO_2_ < 90%^a^	45 (88%)	533 (49%)	<0.001
	Mean HR	158 bpm (150, 168)	152 bpm (145, 160)	<0.001
	Minimum HR	110 bpm (81, 129)	120 bpm (106, 130)	0.008
	Bradycardia^b^	15 (29%)	91 (8%)	<0.001

n (%) or median (Q1, Q3).

*CSTS* Car seat tolerance screen.

^a^at least 10 seconds.

*HR* heart rate.

^b^Bradycardia=HR < 100 beats/minute for >5 seconds.

Among infants who experienced at least one SpO_2_ < 90% event during the CSTS, those that failed had more frequent and prolonged events, and the first event occurred significantly earlier (9 versus 25 minutes from the start of the screen) (Table 4). Bradycardia events were infrequent and short, and occurred later in the CSTS. Infants that failed the CSTS and had at least one bradycardia event had a higher mean HR and a lower HR nadir compared to those that had a bradycardia event but passed the CSTS.

**Table 4:** SpO_2_ and HR characteristics during the CSTS for infants with at least one hypoxemia or bradycardia event.

**Hypoxemia Events**	**Characteristic**	**CST Fail N** = **45**	**CST Pass N** = **533**	**p-value**
	Mean SpO_2_	93.2 (91.5, 95.2)	96.1 (94.6, 97.8)	<0.001
	Minimum SpO_2_	74.0 (68.0, 79.0)	79.0 (73.0, 83.0)	<0.001
	SpO_2_ < 90% Count	11 (4, 19)	3 (1, 7)	<0.001
	SpO_2_ < 90% Total Seconds	276 (82, 690)	62 (22, 164)	<0.001
	SpO_2_ < 90 DPE	26 (17, 37)	16 (12, 27)	<0.001
	Minutes to 1st SpO_2_ < 90%	9 (5, 26)	25 (5, 59)	0.009
**Bradycardia Events**	**Characteristic**	**CST Fail N** = **15**	**CST Pass N** = **91**	**p-value**
	Mean HR	158 (150, 165)	145 (130, 155)	0.002
	Minimum HR	76 (71, 84)	86 (76, 93)	0.048
	Brady Count	1 (1, 2)	1 (1, 2)	0.5
	Brady Total Duration	12 (6, 18)	12 (6, 22)	0.8
	Brady DPE	9 (6, 13)	8 (6, 12)	0.8
	Minutes to 1st Brady	62 (13, 98)	58 (23, 93)	0.9

Median (Q1, Q3).

*HR* heart rate beats/minute.

*Brady* bradycardia <100 beats/minute at least 5 seconds.

*DPE* duration per event (in seconds).

## Discussion

In this large cohort of infants at a single NICU, we found that infants who failed their first CSTS had more oxygen desaturation events in the 24 h period prior to the screen. Many infants that passed the CSTS had short episodes of desaturation and/or bradycardia but those who failed tended to have a greater number, depth, and/or duration of events. Gestational age and NICU length of stay were similar for the pass and fail groups in the entire cohort but in the smaller cohort of infants with vital sign data available for analysis, gestational age was lower and length of stay longer for those that failed the first CSTS. Others have reported CSTS failure rates ranging from 4% to 12% compared to our rate of 4.4%.^1,11–14^ The variability in failure rates could reflect differences in patient populations or in guidelines for screen performance and interpretation.^15^ We did not find differences in pass/fail rates based on diagnosis of severe intraventricular hemorrhage or chronic lung disease requiring home oxygen. We found that GA was similar for patients that passed or failed their first CSTS, which aligns with some published data,^3,16^ but differs from other reports of lower GA^12^ and higher GA^17^ for infants that fail the screen. Again, these different findings are likely due to marked variability in timing and interpretation of the screen both between and within centers.^2,4^ We found that PMA at the time of the first CSTS was not significantly different between pass and fail groups. In contrast, a report of nearly 8,000 infants at 788 level II-IV NICUs found that higher PMA was associated with a lower CSTS failure rate, perhaps indicating that clinicians in those units waited until desaturation events had resolved before performing the screen.^1^

By analyzing granular SpO_2_ and HR data, we found that many infants that passed the CSTS had at least one episode of hypoxemia during the screen, and some had transient bradycardia. Infants with CSTS failure tended to have a greater number, depth, and/or duration of events, consistent with our nursing guideline that suggests that desaturation or decelerations should only be counted as a failed screen if they last more than 20 seconds. We found that in the day prior to the CSTS, infants with screen failure had more desaturation events and slightly lower mean SpO_2_ and, illustrating that although they were judged to be ready for NICU discharge within 2 days, they were more vulnerable to intermittent hypoxemia compared to infants that passed the screen. Some have suggested that the CSTS can identify infants that are not ready for discharge home.^14^ For example, a review of 148 late preterm or term infants in a mother-baby unit who failed the CSTS and were transferred to the NICU reported that many of these infants were found, through NICU monitoring, to have apnea, hypothermia, hyperbilirubinemia and other conditions requiring longer hospitalization.^18^

The AAP recommendation for car seat screening was based on preterm infants’ tendency to have desaturation and bradycardia events and the potential that these would be exacerbated when seated semi-reclined in the car seat, due to hypotonia and airway compromise.^19^ Indeed, in a car motion simulation study of multiple term and preterm infants (median weight 2.5 kg), oxygen desaturation was significantly more common when seated at a 30 degree angle compared to when lying supine, and major desaturation events (SpO_2_ < 85%) were significantly increased during simulated car motion than when infants were in stationary car seats.^3^ The clinical impact of these events is not clear, since another study showed that cerebral oxygenation did not decline during systemic oxygen desaturation spells during the car seat challenge.^20^ In addition to potentially identifying infants with short-term vulnerability, there is limited evidence that cardiorespiratory instability in a car seat may be associated with long-term health concerns, with one case/control study reporting that NICU patients that failed the CSTS were more likely to have asthma and sleep-disordered breathing at age 2–3.^21^ A final potential benefit of CSTS is the opportunity to educate parents on correct placement of their infant with appropriate head support and with the straps and buckle properly secured, and assuring they understand how to place the seat in the car since this has been shown to be challenging.^22^

Some have questioned the need for CSTS, since there is no published evidence that routine performance of the test reduces morbidities or mortality.^23^ A recent Canadian Pediatric Society statement highlights insufficient evidence to support routine use of the CSTS in discharge planning for preterm infants.^24^ An argument against CSTS is that it could contribute to delayed discharge,.^1,25^ In our large NICU cohort the duration of hospitalization was not different between the pass and fail groups, though in the smaller cohort with continuous vital signs available, gestational age was 1 week lower and length of stay 11 days longer, on average, for infants that failed the initial CSTS. Some hospitals have moved away from mandatory CSTS, and in a report from multiple hospitals in California, discontinuation of screening did not lead to increased deaths, 911-triggered transports, hospital readmission for apnea, or other adverse outcomes.^5^ The majority of hospitals in the US continue to follow the AAP recommendation but with great variation in test performance and interpretation. A 2019 survey of neonatologists from around the world found that 72% of those practicing in the US and only a small fraction of those outside the US believed that a CSTS was medically necessary.^26^ A minority of survey respondents indicated they would have equipoise for a randomized clinical trial of CSTS versus no CSTS, but a majority would accept a trial of permissive versus restrictive criteria for CSTS failure. Given the considerable debate over whether and how to perform and interpret a CSTS, and the implications for infant safety and for healthcare resource allocation, it would seem that such a trial would be beneficial.

Strengths of our analysis include a large cohort of NICU infants who underwent CSTS, and our ability to analyze continuous HR and SpO_2_ data before and during the screen for many of these infants. A limitation is that, in a retrospective chart review, we may not have captured all the reasons that an infant was determined to have failed their screen. Also, our NICU testing protocol and nursing guidelines about what constitutes CSTS failure may differ from that of other units, limiting the generalizability of our results. And finally, the fact that some infants passed a repeat CSTS less than 12–24 hours later indicates that the failed screen may have been due to suboptimal positioning or variability in clinicians’ interpretation of the significance of transient decline in HR and SpO_2_.

In the years of this analysis, our unit guideline was to perform a 120-minute CSTS within 2 days of discharge, which led to repeat testing for 130 of our infants that passed the initial screen but had delayed discharge. A small number of those infants failed their second CSTS which aligns with others’ findings of test/retest reliability.^13^ We note that in 2023 our NICU clinical leadership team changed the CSTS protocol for pragmatic reasons, without awareness of the data presented here. Our new protocol requires a 90 min (rather than 120 min) CSTS and does not specify that it must be performed within 2 days of discharge. We anticipate this will simplify NICU discharge without increasing risk.

## Conclusion

In this large single-NICU retrospective review, 4.4% of infants failed their initial CSTS with most passing a repeat screen within a short timeframe, and no significant difference in gestational age or NICU length of stay for the pass and fail groups in the overall cohort. In the 24 hours prior to the CSTS, infants that failed the screen had more and deeper episodes of desaturations and bradycardia compared to those that passed. While the CSTS may uncover positional instability of HR or SpO_2_, more work is needed to find the best way to administer and interpret the screen and to determine whether and how it benefits NICU patients preparing for discharge home.

## Supplementary information


Supplemental Table S1


## Data Availability

The datasets analyzed in the current study are available from the corresponding author on request.
