# Supplementary material for: Car seat tolerance screen failure in the neonatal intensive care unit: clinical, oxygen saturation and heart rate analysis
Source: Pediatr Res. 2025 Oct 27;100(1):257–62. doi: 10.1038/s41390-025-04524-w (PMC12951016; doi:10.1038/s41390-025-04524-w)
Supplement: Supplementary file 1 [file 41390_2025_4524_MOESM1_ESM.pdf]

Supplemental Table S1: Patient characteristics for sub-population of infants with SpO_2 and HR analyzed, based on first CSTS pass/fail

SpO2 and HR Analysis Population

Characteristic	CST Fail N = 51[1]	CST Pass N = 1,085[1]	p-value[2]
Female	20 (39%)	478 (44%)	0.5
Race			0.001
Other/Unknown	0 (0%)	107 (9.9%)	
Black	4 (7.8%)	210 (19%)	
White	47 (92%)	768 (71%)	
Ethnicity			0.5
Non-Hispanic	45 (88%)	985 (91%)	
Hispanic	6 (12%)	100 (9.2%)	
Gestational Age (weeks)	33.0 (31.1, 34.4)	34.0 (31.3, 36.0)	0.056
Birthweight (kg)	1.80 (1.30, 2.47)	2.00 (1.48, 2.56)	0.3
Oxygen at Discharge	4 (7.8%)	76 (7.1%)	0.8
PMA at CSTS	36.77 (35.87, 38.67)	36.98 (35.84, 39.13)	>0.9
Length of Stay (days)	30 (18, 54)	19 (12, 45)	0.034
Severe IVH (Grade III-IV)	1 (2.0%)	20 (1.8%)	>0.9

[1] n (%); Median (Q1, Q3)

[2] Pearson's Chi-squared test; Fisher's exact test; Wilcoxon rank sum test

CSTS=car seat tolerance screen; IVH=intraventricular hemorrhage; PMA=post-menstrual age
